# The Art of Staying Well: How Creative Practice Shapes Personal Recovery Beyond Clinical Remission in Bipolar Disorder

**DOI:** 10.7759/cureus.113461

**Published:** 2026-07-27

**Authors:** Janny P Laurean-Soto, Shoshana Berenzon, Ingrid Vargas-Huicochea

**Affiliations:** 1 Education, Instituto Nacional de Psiquiatría Ramón de la Fuente Muñiz, Mexico City, MEX; 2 Epidemiological and Social Research, Instituto Nacional de Psiquiatría Ramón de la Fuente Muñiz, Mexico City, MEX; 3 Psychiatry and Mental Health, Faculty of Medicine, Universidad Nacional Autónoma de México, Mexico City, MEX

**Keywords:** art, bipolar disorder, euthymia, personal recovery, qualitative research, stigma

## Abstract

Background and objectives: Bipolar disorder (BD) carries a substantial global health burden. While pharmacological treatments effectively achieve clinical remission, functional and personal recovery often remain suboptimal. Although formal art therapy has been explored in psychiatry, the role of daily, organic artistic practice as a lived mechanism for personal recovery in BD is underexplored. This study aimed to analyze how artistic practice influences the personal recovery process from the perspective of outpatients with BD.

Materials and methods: A qualitative study with a phenomenological design was conducted at the outpatient clinic of the Instituto Nacional de Psiquiatría Ramón de la Fuente Muñiz (INPRFM) in Mexico City. A non-probabilistic, purposive sample of typical cases technique was utilized to recruit adult outpatients with BD type I who were in syndromal recovery (euthymia) and actively engaged in artistic practices. Data were collected through semi-structured interviews and analyzed using thematic analysis based on the personal recovery model. Methodological rigor was ensured by adhering to the Consolidated Criteria for Reporting Qualitative Research (COREQ) guidelines.

Results: Nine participants (seven women and two men) were included until information saturation was reached. Artistic practice functioned as a multifaceted catalyst for personal recovery. Core themes identified included the redefinition of personal identity and the mitigation of self-stigma, the cultivation of hope, and the discovery of new life meanings. Additionally, artistic engagement fostered a profound sense of responsibility toward self-management and treatment adherence, while simultaneously promoting spiritual, cognitive, and social connectedness.

Conclusions: Daily artistic practice extends beyond mere symptom management, acting as a vital component of personal recovery in BD. Recognizing and integrating patients' artistic interests into clinical discussions may enhance therapeutic alliances, combat internalized stigma, and promote holistic well-being in this population.

## Introduction

Bipolar disorder (BD) is a chronic and severe mood disorder characterized by recurrent episodes of mania, hypomania, and depression, carrying a substantial global health burden [1]. The primary goal of pharmacological and psychosocial interventions in BD is to achieve syndromal recovery, defined as the remission of acute symptoms and the maintenance of euthymia [2]. However, clinical remission does not invariably translate to functional or personal recovery. Many patients continue to experience psychosocial impairment, cognitive deficits, and a diminished quality of life, highlighting the limitations of a strictly biomedical approach to the disorder [3].

In recent years, the conceptualization of recovery in severe mental illness has shifted from a purely clinical paradigm to a personal recovery model. According to Andresen et al., personal recovery is a deeply subjective, non-linear process that involves finding hope, redefining one's identity, building a meaningful life, and taking responsibility for one's own wellness, regardless of the presence of residual symptoms [4]. Standard psychiatric interventions, while essential for mood stabilization, often fail to address these existential and psychosocial dimensions. Consequently, there is a pressing need to identify adjunctive, patient-driven strategies that facilitate personal recovery and improve long-term outcomes in BD.

The relationship between BD and creativity has been widely documented, with individuals exhibiting higher rates of artistic engagement and creative professions [5]. While structured art therapy has shown efficacy in reducing depressive and anxious symptoms across various psychiatric populations [6], the organic, daily practice of art as a self-directed coping and recovery mechanism remains poorly understood. Engaging in artistic activities - whether through visual arts, literature, or music - may provide a unique avenue for emotional sublimation, cognitive restructuring, and the externalization of internal experiences. Furthermore, art can serve as a powerful tool for narrative reconstruction, allowing individuals to reframe their illness experience and challenge the pervasive stigma associated with severe mental illness.

Despite the anecdotal and historical associations between BD and artistic expression, empirical qualitative evidence detailing how daily artistic practice specifically influences the personal recovery model in euthymic patients remains scarce, with most existing literature focusing on structured clinical interventions rather than organic, self-directed practices [7,8]. Understanding this phenomenon is crucial for developing more comprehensive, patient-centered care models. Therefore, this study aimed to explore and analyze the role of artistic practice in the personal recovery process of outpatients with BD type I, focusing on their subjective experiences regarding identity, meaning, hope, and treatment responsibility.

## Materials and methods

Study design and setting

This was a qualitative study utilizing a phenomenological design, an approach that focuses on describing and understanding a specific phenomenon through the individual lived experiences of the participants, capturing the meanings they attribute to it [9]. This descriptive phenomenological approach followed Husserlian principles of bracketing (epoché), in which the research team set aside prior clinical assumptions about BD and artistic practice during data collection and analysis; transcripts were subsequently organized following Colaizzi's method of extracting significant statements, formulating meanings, and clustering these into themes [10].

The study was conducted at the outpatient affective disorders clinic of the Instituto Nacional de Psiquiatría Ramón de la Fuente Muñiz (INPRFM) in Mexico City, a tertiary psychiatric referral center, between February 2022 and November 2023.

Study participants and sample size

Participants were recruited using a non-probabilistic, purposive sample of typical cases technique [9]. The inclusion criteria were: adults aged 18 years or older, a confirmed clinical diagnosis of BD type I according to the Diagnostic and Statistical Manual of Mental Disorders, Fifth Edition, Text Revision (DSM-5-TR) criteria [11], current syndromal recovery (euthymia) sustained for at least six months, and active engagement in at least one artistic practice (either professionally or as an amateur/hobbyist) for a minimum of one year prior to the study. The psychiatric diagnosis and the sustained euthymia were confirmed through review of the clinical chart and corroborated by the treating psychiatrist.

Patients were excluded if they presented with active psychotic symptoms, severe cognitive impairment that precluded informed consent, active substance use disorders, or any comorbid psychiatric condition that significantly impaired their ability to participate in an interview. Active substance use disorder was determined through clinical assessment by the treating psychiatrist rather than through laboratory or toxicological testing, consistent with standard outpatient recruitment procedures for qualitative research; this is discussed as a limitation below.

The sample size was not predetermined; instead, interviews were conducted until information saturation was achieved, defined as the point where no new themes or significant information emerged from subsequent interviews [12].

Study tool and sampling procedure

Data were collected through semi-structured individual interviews, a methodological act that prioritizes the free discourse of each participant guided by the interviewer [13]. The interview guide was developed based on the theoretical framework of the personal recovery model (encompassing hope, identity, meaning, and responsibility) and the conceptualization of art as a medium for emotional expression. During the interview, priority was given to the free speech and free discourse of each participant, guided by the interviewer to obtain information that allowed exploring the participants' illness narratives, their relationship with art, and their perceptions of how art influenced their mood stability, self-concept, and daily functioning.

Interviews were conducted either in a private, quiet office at the institute or via secure, institutional videoconferencing platforms, depending on the participant's preference and prevailing public health guidelines. Each session lasted between 90 and 120 minutes. The principal investigator, a psychiatrist trained in qualitative research, conducted all interviews to ensure clinical containment in the event of emotional distress.

The final interview guide is provided in the Appendices. To limit confirmation bias associated with the a priori categories of the personal recovery model, the guide relied on open, non-leading prompts, and interviewers were instructed to explicitly invite accounts of difficulty or ambivalence associated with artistic practice. The interviewing researcher was not the treating psychiatrist for any participant.

Data analysis

All interviews were audio-recorded and transcribed verbatim. The qualitative data were analyzed using thematic analysis. Transcripts were read multiple times to achieve data immersion. Two members of the research team independently generated initial codes from a subset of transcripts; codes were compared, and discrepancies were resolved through discussion, with a third author arbitrating where consensus could not be reached. Initial codes were subsequently grouped into broader themes aligned with the a priori categories of the personal recovery model, as well as allowing for emergent themes. The analysis was conducted manually to ensure a deep, contextual understanding of the narratives. Direct quotations were selected to illustrate the core themes and enhance the credibility of the findings.

Methodological rigor

To ensure methodological rigor and transparency, this study adhered to the Consolidated Criteria for Reporting Qualitative Research (COREQ) guidelines [14]. The research team maintained reflexivity throughout the data collection and analysis phases, acknowledging their clinical backgrounds and potential biases. Detailed audit trails of the coding process and thematic development were preserved to guarantee the confirmability and dependability of the findings.

Ethical considerations

The study protocol was reviewed and approved by the Institutional Ethics Committee of the INPRFM. All participants provided written informed consent prior to enrollment. The study was classified as minimal risk. Protocols were established to manage potential emotional distress during the interviews, including immediate clinical containment by the investigator and referral to the institute's continuous psychiatric care unit if necessary. Participant confidentiality was strictly maintained through the use of alphanumeric codes, and all digital data were stored on password-protected institutional servers.

## Results

Participant characteristics

A total of nine participants completed the study until information saturation was reached. The sample comprised seven women and two men, aged 26 to 57 years. All participants had a confirmed diagnosis of BD type I and were in a state of syndromal recovery (euthymia), with durations of stability ranging from 1.5 to 10 years. Regarding their artistic engagement, eight participants were professional artists (working in plastic arts, visual arts, photography, and dance), while one participant, a medical doctor, engaged in art empirically as a daily hobby. Most participants practiced more than one artistic discipline, frequently combining visual arts with literature or music. The sociodemographic and clinical characteristics of the sample are detailed in Table 1.

**Table 1:** Sociodemographic and clinical characteristics of the participants (N=9)

Characteristic	n (%)
Sex	
Female	7 (77.8)
Male	2 (22.2)
Age group (years)	
26-32	4 (44.4)
44-45	3 (33.3)
56-57	2 (22.2)
Primary artistic discipline	
Plastic/visual arts and photography	8 (88.9)
Dance	1 (11.1)
Years of euthymia	
1.5-3 years	3 (33.3)
6-7 years	2 (22.2)
9-10 years	4 (44.4)
Previous suicide attempts	
None	6 (66.7)
One or more	3 (33.3)

Categories

Illness Perception and the Mitigation of Self-Stigma

The majority of the participants reported a significant delay in receiving the correct BD diagnosis, often enduring years of misdiagnosis with unipolar depression or brief psychotic disorders. However, receiving the definitive diagnosis was largely experienced as a liberating event that allowed for treatment optimization. Artistic practice played a pivotal role in the acceptance of the illness. Participants frequently referenced historical artists, such as Vincent van Gogh, to contextualize their own emotional intensity and creative drive, reframing the disorder not as a deficit, but as an intrinsic component of their identity.

Furthermore, art served as a powerful mechanism to dismantle internalized stigma. Several participants described using their artistic platforms - whether through autobiographical writing, theater, or visual exhibitions - to publicly disclose their diagnosis and educate society. This active engagement in anti-stigma advocacy shifted their self-perception from one of shame to one of empowerment. Participants also normalized their condition by drawing parallels with other chronic medical illnesses, with one participant noting, "The illness is like diabetes or hypertension; it is a chronic condition, and that is how I see it now."

Redefinition of Identity and the Search for Meaning

Artistic engagement was universally described as a core pillar of the participants' identity. The absence of creative practice was associated with feelings of emptiness and a loss of purpose. The creative process facilitated profound self-knowledge and improved self-esteem. For instance, one participant who experienced significant weight gain as a side effect of psychotropic medication found that participating in a nude drawing workshop allowed her to reconnect with and accept her body, restoring her self-confidence.

In terms of finding meaning in life, the practice of art catalyzed the adoption of new values, particularly solidarity, empathy, and environmental consciousness. Participants reported that their artistic work was no longer just a source of income, but a vehicle for social transformation. By sharing their lived experiences through their art, they found a renewed sense of purpose, actively contributing to the mental health awareness of their communities.

Hope, Emotional Regulation, and Cognitive Benefits

Participants identified art as a fundamental tool for maintaining hope and affective stability. They expressed a strong conviction that their artistic practice directly prevented mood relapses. When describing periods where they had to stop creating, participants noted the rapid onset of dysphoria, irritability, or depressive symptoms. The creative act was described as a grounding mechanism that provided immediate emotional regulation and sublimation of negative affects such as anger, grief, and anxiety. One participant explained, "When I start working on a sculpture, I immediately return to my center. Art is the most stable part of my life."

Additionally, emergent cognitive benefits were identified. A participant who practiced dance reported that the rigorous memorization of choreography and the demand for sustained attention significantly improved her cognitive functioning and memory, particularly during the recovery phases following severe acute episodes.

Responsibility in Recovery and Therapeutic Alliance

The discipline required by artistic practice translated into better self-management and healthier lifestyle habits. Participants reported strict adherence to sleep hygiene, balanced diets, and the avoidance of psychoactive substances, recognizing that these routines were essential for both their artistic productivity and their mood stability.

Interestingly, while art provided the emotional and existential motivation for recovery, the participants emphasized that their pharmacological adherence was primarily driven by a strong therapeutic alliance with their psychiatrists and comprehensive psychoeducation. The introspection fostered by art complemented this medical adherence, allowing participants to view their medication not as a restriction, but as a necessary tool to preserve their creative capacity. As one participant stated, "The medication is not something that harms me; it is for my well-being, allowing me to do my things and stay healthy."

Spiritual and Social Connectedness

The practice of art profoundly enhanced the participants' interpersonal relationships and spiritual well-being. Creating art was frequently described as a ritualistic or spiritual experience that fostered a deep connection with themselves, nature, and a higher power. Socially, art acted as a bridge, strengthening bonds with family members and friends who shared these creative interests, and expanding their social networks through workshops and exhibitions. This connectedness was perceived as a vital protective factor against the isolation often experienced in severe mental illness.

Table 2 summarizes representative verbatim quotations illustrating the core themes identified in participants' narratives of their personal recovery journey.

**Table 2:** Representative participant quotations illustrating the role of artistic practice in personal recovery

Category	Representative quotation	Participant
Illness perception and normalization	"The illness is just like diabetes or hypertension; it is a chronic condition, and that is exactly how I see it now."	P4
Mitigation of self-stigma and advocacy	"I visually narrate all my lived experiences... I express them in my own visual language. The intention has always been to talk about this topic openly, so people understand that these things happen and that it is more common than we think."	P5
Identity and historical connection	"The first painter I encountered was Vincent Van Gogh. After finding out he was sick just like me, I understand why I am the way I am, why I paint the way I do, and why I am so passionate about certain things."	P2
Redefinition of identity and meaning	"It is a part of me. If I am not creating, I sometimes feel that life simply has no meaning."	P7
Self-esteem and spiritual security	"I had a difficult childhood... I feel that is where my low self-esteem came from. But I have been working on it, and art helps me. I feel that through art, you gain a sense of security, but one that comes from the spirit."	P3
Hope and emotional regulation	"When I started working on that sculpture, I realized I was returning to my center, that I was stable. Art is truly the key... it is the most stable part of my life."	P1
Cognitive benefits (Dance)	"It helps my memory immensely. When I am just discharged from the hospital and my memory is not very good, it helps me tremendously because I have to learn every single step. You have to be completely present and focused in that moment."	P8
Responsibility and pharmacological adherence	"The medication is not something that harms me; it is for my own good. It is not harmful, on the contrary, it is for my well-being, so I can do my things and stay healthy."	P6
Therapeutic alliance and psychoeducation	"The doctors who have treated me are very kind; they listen to you and answer all your questions. They are incredibly disciplined about ensuring you don't relapse, they are always there. I truly appreciate that."	P9
Spiritual connectedness	"It makes me feel free when I do it, and in that moment, it feels almost religious. Like when someone enters a church and prays. When I am painting, I concentrate, and I am in a kind of ritual that fills my spirit."	P4
Social connectedness and family bonds	"It has brought me much closer to my family. For my recent recital, my grandfather, my grandmother, my uncle, and my little cousin all came to watch me."	P8

## Discussion

This qualitative study explored the lived experience of artistic practice among euthymic outpatients with BD type I, mapping these experiences onto the personal recovery model. The findings reveal that daily, organic artistic engagement extends far beyond a mere leisure activity. Participants described it as a multidimensional contributor to personal recovery, one they associated with the redefinition of identity, the mitigation of self-stigma, the cultivation of hope, and the assumption of responsibility for one's own wellness.

The redefinition of identity is a critical component of personal recovery, as severe mental illness often fractures an individual's sense of self [15]. This metacognitive quality - the capacity to step back and observe one's own emotional and creative process - recurred across several narratives, consistent with broader accounts of reflective self-awareness in recovery-oriented frameworks. In our study, participants successfully integrated the diagnosis of BD into their life narrative, largely facilitated by their artistic identity. By identifying with historical figures who exhibited similar mood fluctuations and creative brilliance, participants reframed their illness from a stigmatizing label to a source of empathy and creative depth. This aligns with the concept of narrative reconstruction, where individuals use creative expression to challenge internalized stigma and reconstruct a positive self-concept [16]. The use of art as a public platform for anti-stigma advocacy further empowered the participants, transforming their vulnerability into a tool for social education and personal growth [17].

The cultivation of hope and the finding of meaning are deeply intertwined in the recovery process [18]. Our participants described art as an indispensable anchor for emotional regulation and mood stability. The fear of losing their creative capacity if they ceased practicing art underscores how deeply intertwined the artistic drive is with their psychological equilibrium. This organic sublimation of emotions provides a safe space for processing complex affects, which is particularly relevant for individuals with BD who may struggle with emotional identification during subsyndromal phases [19]. Furthermore, the spiritual and existential connectedness fostered by art resonates with previous findings indicating that creative practices can enhance spirituality, providing a sense of purpose that transcends the mere absence of symptoms [20].

A particularly nuanced finding of this study relates to the responsibility in recovery and treatment adherence. While the discipline derived from artistic practice positively influenced lifestyle modifications and self-care routines, the participants explicitly attributed their pharmacological adherence to the quality of the therapeutic alliance and psychoeducation received from their mental health providers. This highlights a crucial clinical distinction: while art provides the existential motivation and emotional containment necessary for recovery, it does not replace the need for a robust, empathetic medical alliance to ensure biological stability [21]. The introspection fostered by art likely enhances the patient's insight, making them more receptive to psychoeducation and more proactive in their self-management. This is consistent with the broader psychotherapy outcome literature, in which the therapeutic alliance ranks among the most robust predictors of treatment outcome across modalities [22].

Clinical implications

The findings of this study carry significant implications for clinical practice. Psychiatrists and mental health professionals should routinely inquire about their patients' creative interests and hobbies, recognizing them not as trivial pastimes, but as vital coping mechanisms and pillars of personal recovery. Integrating this knowledge into clinical discussions can strengthen the therapeutic alliance, validate the patient's subjective experience, and provide a holistic understanding of their recovery journey. Whether these findings extend to community-based artistic programs serving amateur artists or individuals without formal artistic training remains an open question; given that eight of nine participants in this study were professional artists, prospective evaluation in more heterogeneous populations is needed before community-based artistic programming can be recommended as a patient-driven strategy for social inclusion and stigma reduction [23].

Limitations

This study has several limitations. The sample size is small, which is typical for qualitative phenomenological research, but it limits the transferability of the findings, and the cross-sectional, single-time-point design cannot establish whether the reported redefinition of identity and sense of stability persist independently of sustained artistic engagement. The sample is predominantly female and heavily skewed toward professional artists - eight of nine participants derived part or all of their livelihood from artistic work - which constitutes a significant selection bias: individuals with higher cognitive reserve or socioeconomic status might have greater access to formal artistic training, and these findings should not be assumed to generalize to people with BD who engage with art casually or without formal training. Because participants were purposively selected for sustained, self-identified beneficial artistic practice, the near-uniformly positive tenor of the findings likely reflects this selection rather than the absence of adverse or ambivalent experiences - such as sleep disruption, perfectionism, or occupational pressure - among people with BD who practice art more broadly; this is a direction for future work using a broader interview protocol not anchored exclusively to the personal recovery model. Additional methodological limitations include the retrospective and self-reported nature of the data, the potential for social-desirability bias given that a psychiatrist conducted the interviews, the possibility of interviewer influence on disclosure, a theory-driven confirmation bias arising from an interview guide and coding frame built around the a priori categories of the personal recovery model, and the consequent inability to determine the direction of association between artistic practice and recovery - that is, whether art facilitates recovery, recovery facilitates renewed artistic engagement, or the two influence one another reciprocally. Substance use was excluded on clinical rather than toxicological grounds, which may have allowed under-disclosed use to go undetected. Finally, the study was conducted in a single tertiary psychiatric center in Mexico City, which may reflect specific cultural and institutional contexts. Future research should explore the role of art in personal recovery among more diverse populations, including amateur artists, individuals from lower socioeconomic backgrounds, and those with comorbid substance use disorders, using longitudinal designs capable of establishing temporal precedence.

## Conclusions

In this group of predominantly professional artists with BD, daily artistic practice was perceived as a multidimensional contributor to personal recovery. Participants associated their artistic engagement with the redefinition of identity, the mitigation of self-stigma, emotional regulation, and spiritual and social connectedness, describing these as central to building a meaningful life beyond clinical remission; whether these perceived gains are attributable to artistic practice itself, to the broader recovery process, or to their reciprocal influence cannot be determined from this cross-sectional design. Pharmacological adherence, by contrast, was attributed by participants primarily to a strong therapeutic alliance and comprehensive psychoeducation rather than to artistic practice itself. Mental health professionals may consider inquiring about patients' artistic engagement as part of a holistic, patient-centered approach to recovery, while recognizing that the evidence for this practice, at present, rests on the perceptions of a small and highly selected group of participants.

## Appendices

Interview guide

"The Art of Staying Well: How Creative Practice Shapes Personal Recovery Beyond Clinical Remission in Bipolar Disorder"

The semi-structured interview guide was organized around five domains derived from the personal recovery model, exploring participants' experience of artistic practice in relation to hope, identity, meaning, responsibility, and personal growth. Interviewers used these domains as thematic prompts rather than a fixed question script, allowing participants' free discourse to guide the depth and order in which each area was explored.

1. Hope - Perception of art as a promoter of hope in recovery from affective disorders

The patient's confidence in achieving their proposed goals, and confidence in improving their health or sustaining a good, ongoing state of well-being.

2. Identity - Positive self-perception and patient identity

The participant's sense of who they are following the bipolar disorder diagnosis and following their engagement in artistic practice.

3. Meaning - Perception of how art has helped patients with bipolar disorder find new meaning in life

The emergence of new goals, values, foundations, motivations, and life purposes.

4. Responsibility - Patient responsibility in their recovery

Illness awareness, pharmacological adherence, adherence to follow-up care, self-care, self-management of well-being, and commitment to treatment.

5. Growth and connectedness - Perception of the promotion of personal growth through art

Empowerment and self-determination, and growth and functioning across family, social, occupational, and spiritual domains.
